# Clinical Lectures on Surgery

**Published:** 1855-11

**Authors:** 


Art. III.—*Clinical Lectures on Surgery.* By M. Nelaton. From Notes taken by Walter F. Atlee, M. D. Philadelphia: J. Lippincott & Co. 1855. 8vo. pp. 755.

---

Dr. Atlee is a very industrious translator. Only a year ago he laid his countrymen under obligations by translating his notes, which were very closely and faithfully taken, of M. Bernard's Lectures on the Blood. He has now rendered into English Nélaton's Clinical Lectures on Surgery. The volume contains the publication of notes taken, during a period of three years,

1451-2-3-4, from the remarks the French Surgeon made upon cases admitted into the Clinical Hospital of the Faculty of Medicine, of Paris.

We ourselves have had some experience in this kind of labor, and most cordially say of Dr. Atlee that he is an admirable note taker, and much the best translator of the French who has yet appeared on this side of the Atlantic. And what, we think, is not less to his praise, he has, though a *Philadelphian*, forborne doing any great amount of annotating. What he has done in that way is judicious and—brief.

The course which Dr. A. has adopted in regard to the arrangement of the cases, has been to class under the same head those in which the same pathological lesion existed—a course obviously incomparably superior to its opposite, an example of which is seen in Prof. Bedford's Obstetric Clinique. The copious table of contents and the well-arranged index make it a work of easy reference.

Since M. Nélaton can but be regarded as one of the leading minds of the French School of Surgery, his lectures are entitled to most favorable consideration. They will amply repay an attentive perusal. They are, in our opinion, *model Clinical Lectures*, and we sincerely wish that among their many readers in this country they may fall into the hands of the teachers of both Clinical Medicine and Surgery. The "Good morning, my good woman," style does not appear in M. Nélaton's book. We are spared any elaborate sermons on virtue, morality and the like, and have, instead, a plain, straight forward, well told narrative of cases, with judicious and practical comments thereon.

An idea of the general style of the work, and the manner in which the American editor has discharged his task, may be obtained from the following cases selected from the chapter on Cancerous Affections of the Bones. It is hardly necessary to remark that M. Nélaton has, perhaps, greater celebriety in this particular department of surgical pathology than in any other:—

"*Of the Femur.*—November, 1853. A man, forty years of age, quite emaciated, but not in marasmus; his condition still admitted of a serious operation. He had been sent to the hospital as affected with fungosities of the synovial membrane at the knee.

"Of the antecedents of the patient only one need be mentioned, and that with reserve. He said that his father died at the age of fifty-nine, from an ulcer of the leg, after having suffered a great deal from it. Now, it cannot be affirmed that an ulcer which caused great suffering, was an ulcerated cancer, but it is possible that it was so.

"In the month of December, 1852, he felt the first symptoms of the affection, what he called *engourdissement*, numbness, in the left knee, and feebleness in the left lower extremity. When examined as to the sensation, he said at first there were pains like flashes of lightning shooting through the part, very rare, but afterward more frequent; the pain, when he came to the wards, was a dull, heavy pain. In the month of February following, he noticed a tumor on the internal lateral portion of the femur, above the condyle, as large as the egg of a chicken, and its great diameter in the axis of the limb; it increased in size, and at last gained the anterior, then the external part of the thigh, and at the same time the popliteal space was invaded. Moreover, this tumor was very hard at first; the patient had sought to move it, but it was very firmly fixed. The man was very intelligent, and had observed very well the progress of his disease.

"At the time of his entering the tumefaction was very considerable, above all at the superior part of the knee, or, more properly speaking, the inferior portion of the femur; this tumefaction was quite regularly uniform. On the exterior the skin was marbled, in some places from small ecchymoses, in others from blood-vessels which could be seen through the thinned integument; in the upper part, on the thigh, the veins were enlarged, and at parts corresponding to these veins, the tumor felt as if grooved. As to the consistence, at the internal part, where it had commenced, the softness was remarkable; there, there was fluctuation, what is called *false* fluctuation, a bad term, for the fluctuation was as frank here as if there had been a collection of pus. In other parts of the tumor the softness was not so great. By attention, on leaving the hand applied, uniform pulsation could be felt. In the popliteal space, the *internes* thought they could perceive a *bruit-de-souffle;* M. Nélaton himself could not hear it, probably from the noise in the ward when he made his visit.

"Without going further the diagnosis of this disease could be circumscribed to three affections. 1. White swelling, the kind formed by fungosities of the synovial membrane; 2. A vast hydrarthrosis with thickening of the walls; or 3. A cancerous affection. M. Nélaton has seen errors made with all these three affections, and by intelligent men, well up to the times as regards the science of surgery.

"Fungosities of the synovial membrane, in some cases, present the same appearance that this one did, giving to the touch a feeling of softness and of fluctuation. Again, another circumstance is this, that some white swellings of this kind present pulsations, and the surgeon must take care not to be deceived by them. There were many other reasons for refusing to admit this disease to be a white swelling, but M. Nélaton, in mentioning them, limited himself to the chief, the origin: the tumor has commenced at some distance from the joint and finished by covering it; again, at the beginning it was hard: there is nothing like that in tumors formed by fungosities; again, in some places it was hard, in others soft; when the synovial is affected, all is equally soft. Without hesitation, then, the supposition of its being this affection would be rejected.

"Between the other two affections the diagnosis is more easy, yet as the error has been committed of confounding them, attention should be called to the possibility of it. A man had been in the wards but a short time before, who had entered for cancer, and the affection had been proved to be only a vast hydrarthrosis. In such cases all the bonds of union at the articulation are loosened, and the limb turns in every direction; again, they are of very long duration, and it is only when very old that the mistake could be made. In this present instance the tumor had only commenced the preceding February.

"This disease was, therefore, a cancer. Where originally developed was a superfluous affair, so far as diagnosis and treatment were concerned, but M. Nélaton thought that very probably it was a cancer developed in the bone.

"Some cancers begin in bone, and replace, so to speak, the osseous tissue; you find a loss of compact tissue filled up by cancer. In the neighborhood of this loss of substance the bone does not appear to have undergone any alteration. This variety, it may be added, is often seen in the condyles.

"In a second variety, or a second form of cancerous affection of the bones, the bone is pushed before the cancerous mass as it is developed, in place of being destroyed, as in the first form, just spoken of. It is difficult to account for this, but observation seems to show that in the first form the cancerous tissue is in immediate contact with the osseous, while in this there is a fibrous envelop around it. The bone, as it gradually yields, becomes thinner and thinner, and forms at last a very thin, very friable shell around the cancerous mass (spina ventosa of the books).

"A third form resembles this second very much. When the tumor is cut open, a large number of cells are seen, very irregular in their forms and dimensions, and filled by cancerous tissue; it looks as if there were a considerable rarefaction of the bony tissue, in the cells of which the cancerous matter was deposited (osteosarcoma).

"A fourth form is very rare; from a bone a great number of bony needles arise, all, generally, in the same direction for a certain distance, and then a mass of them in another direction; all are plunged in a cancerous mass enveloping the whole. These excessively fine bony prolongations, which might be compared to hairs implanted upon the surface of the bone, are sometimes one or two inches long. Of this variety M. Nélaton showed a specimen in the lower jaw-bone, amputated by M. Maisonneuve.

"In this patient the disease was believed to be this fourth form, developed on the surface of the bone. It very often happens, as was supposed to be true here, that the articulation is not invaded.

"It is important, in speaking of the development of these tumors, to remark that they never invade the cartilaginous tissue; when they have had their origin near the articular extremity of a bone, the diarthrodial cartilage is found intact in spite of the complete degeneration of the epiphysis supporting it. When a joint is invaded, it is by the extension of the disease to a point where the bony tissue is only covered by the periosteum and the synovial membrane.

"What was to be done in this case? In a few months, if left alone, the patient would die from exhaustion. The inguinal ganglions were intact; if even they had been somewhat larger than usual, that would not have been a reason for supposing them to have been invaded by the disease, for leeches and irritating external applications had been made to the knee, before the patient came to the hospital, under the idea that the affection was one of fungosities of the synovial membrane. It was necessary to operate to remove the disease, and the only operation to be performed for this purpose was the amputation of the limb. In regard to the method of doing this, a very oblique incision would be made, in order to take the whole flap for covering the stump from the anterior part of the thigh. This is done in order to avoid a posterior cul-de sac, where the liquids afterward effused can sojourn.

"The incision which M. Nélaton made around the limb was very oblique, as he had said; the anterior portion of the flap, which was of sufficient size to cover the whole bleeding surface, was fastened to the inferior portion by means of some sutures and adhesive strips.

"In order to examine the parts amputated, an antero-posterior section was made, dividing the soft parts and the bone. The tumor was found to have developed itself upon the exterior of the femur; it was the form of cancer of the bone described above, as composed of osseous needles, the whole surrounded by cancerous production. The respect for the diarthrodial cartilages was also noticed; the articulation was free from disease. M. Nélaton added a few words to what he had already said as to the march of these tumors; he has seen a great number of them, and has observed that there is a much smaller chance of a return of the disease than when cancer attacks other portions of the body; the affection demands amputation, but very often the result is durable.

"As to the patient, at first he got along very well. The day after the operation, something was given him to eat, for he was doing very well, and he asked for it. On Monday (the operation was performed on Friday) he complained a little, but he had no chilliness, and it was supposed to be owing to his position in the bed. The stump, at the same time, was somewhat swollen and rather smooth and shining, but this was not objected to by M. Nélaton; he said that he had noticed that those patients who experience some apparently unfavorable symptoms on the part of the stump itself, are those who are least affected with the symptoms he dreads so much, of purulent affection. On Monday night the patient had a chill; on Tuesday he had no repetition of it; but it was a symptom of purulent affection that caused much anxiety. The wound was washed with tincture of iodine, and good diet and strong wine were ordered for the patient.

"On Saturday the man died, after presenting all the symptoms of purulent infection; there were signs of metastatic abscesses in the lungs, and he had complained, above all, of a stitch in the side near the heart; he had spit some blood. At the examination of the body, an abscess was found in the lungs near where the man had complained of pain; on opening it pus was found, and around it there was a peripheric pneumonia. Scattered through the lungs were found small cretaceous masses, and what is remarkable, they were enveloped in pus.

"M. Nélaton was very much affected by the fatal result in this case; things had come to such a point, he said, that really he was forced to ask himself the question, whether, morally, he ought to undertake such operations in his hospital; and it was not the only one in Paris where the mortality was so frightful; radical reforms were needed in all. Of all the cases operated upon, by amputation, in the preceding twelve months, only two had been saved.

"Upon the anatomical pieces, in this case, an instructive experiment was made. Dupuytren, and his school, teach that a ligature should not be applied to an artery in a suppurating wound. M. Nélaton took a fine thread, and the vessel could not be cut by it, though it had remained bathed in suppuration for more than a week. *Arteries in suppurating wounds may be tied;* they can support a ligature as well as any others; the two coats are divided by the thread, and the third resists, as it always does.

"December, 1853. A man, fifty years of age, quite robust, from the country, came to the hospital, for an affection of the knee. Of the antecedents, in this case, M. Nélaton did not speak; he thinks it best, he said, not to encumber his remarks too much with what is, most generally, useless.

"The patient said that three years before a small tumor had formed at the internal face of the knee. When first noticed it was as small as the end of the finger; and when asked to point out the spot where it was situated (for it is of importance to know this), he indicated, as well as could be judged in the existing condition of things, the lower part of the internal condyle of the femur. At first this tumor increased slowly in size; in the course of the first twelve months reaching the volume of a hen's egg; since then, its growth had been more rapid; as a reason for which, he assigned several falls and contusions, but these M. Nélaton thought no importance was to be attached.

"At the time he came to the hospital, at the internal portion of the right knee was a tumor six inches in length, and nearly five in breadth, from one side to the other. It turned about half around the knee, extending from the internal border of the patella around to the biceps muscle. Upwards it extended four inches above the line between the femur and the tibia, and it went down over the tibia as far as where the sartorius is inserted. In many places the fingers could be passed under this tumor. The very greatest pains were taken to see if it only covered the tibia, or came out of it; for some time M. Nèlaton believed that it arose from that bone, but afterwards he concluded not. The first question to be settled, of course, was whether the movements of the tibia were communicated to the mass. Although the tumor moved with the bone, yet it seemed as if there did not exist a perfect correlation in the movements; when the leg is flexed on the thigh, a slight rotatory movement can be impressed upon the leg, and it seemed, here, as if this could be executed without disturbing the tumor. Again, the prolongation of the tumor in the popliteal space was contrary to such a supposition, for such a prolongation would not come from a tumor arising from the anterior part of the tibia. The tumor, therefore, had its origin from the internal face of the condyle of the femur.

"The integrity of the articulation, seen in this case, is what is usually observed.

"The mass was lobulated; in some places it felt as if there were many tumors agglutinated together. It did not have the same consistence everywhere; in some parts, above all at the circumference, it was firm; in the center it was softer, and then at the internal face of the condyle, where the patient had first observed it, it was evidently fluctuating. There are some *tissues* which are fluctuating; a testicle in its physiological state is fluctuating; and there are pathological tissues which are fluctuating.

"At the commencement the patient only had a feeling of numbness about the tumor; afterwards he had lancinating pains; of late the pains had increased, and, at the same time, the skin had become rosy and smooth.

"This tumor, M. Nélaton said, was an ancephaloid tumor; one in which micrographs would very probably not find what they esteem to be cancerous elements, but those found in fibro-plastic tumors.

"If operated upon the chances were very great that it would not return; it was not certain, but very probable. If not operated upon it would become larger and larger, the skin would be perforated; ten days after that the ulceration would be frightful, with hemorrhage and sanious exudation, and the patient would rapidly sink. In six or eight months, if abandoned to himself, M. Nélaton thought, the man would die.

"The only operation to be performed in the case was the amputation of the thigh; the extirpation of the affection was not to be thought of, for the roots would be left in the bones; it was impossible to remove it without removing the bone itself. But amputations succeed horribly in this hospital, M. Nélaton said, and he believed they did no better in the others in Paris, only the surgeons would not confess it. In addition, at that time, the cholera was attacking the patients. The best advice he thougbt he could give to the man was, to go back to his native place, and to persuade the physician of the village to remove the limb for him.

"The preceding case of cancer of the bone was supposed to be one of the third form of that affection, in which there seems to be a considerable rarefaction of the osseous tissue, in the cells of which the cancerous matter is deposited. At his next lecture, M. Nélaton brought a fresh pathological specimen, showing, he said, another form, in which the cancerous matter penetrates into the bone, and causes a loss of osseous substance.

"This specimen was an amputated thigh from a patient operated upon in his private practice. The patient was a young man twenty-two years of age, who had been treated for a syphilitic affection of the upper part of the tibia, till M. Ricord and M. Nélaton were called in, in consultation, when they recognized the affection to be a variety of cancer of the bone. The disease had then lasted only three months, and already the bone could be bent in all directions, as if formed of a soft, elastic substance. The pains were so intense that the patient had not slept for thirty days. The tibia only was affected, but in the most superior portion.

"The amputation of the thigh had been practiced as low down as possible, as it is better for an artificial limb, and also for the chance of cure. The incision in the soft parts could be seen to have been made immediately above the patella.

"Proceeding to the examination of this specimen, when the knee-joint, whose movements had been unaffected, was opened, the articulation was found intact; no disease had penetrated into it. If some had been found in the articulation, it would have been propagated by parts of the tibia not provided with cartilage, which prevents the extension of the cancerous affection. When a vertical section of the tibia was made with a saw, the affection was found not to be cancerous, but one of *erectile tumor of the bone.* In the case of this young man, there had never been any pulsations, nor any souffle, and this had made M. Nélaton believe that the tumor could not possibly be vascular, but must necessarily be encephaloid. The diseased portion of the tibia, when the bone was thus sawn vertically in half, appeared to be about five inches long, in the direction of the long diameter of the bone, and three and a half wide. The affection, M. Nélaton said, was between erectile tumor and what is called multilocular cyst of bones, in which sometimes the contained fluid is sero-sanguineous. In the work of Scarpa, an exact representation of what was here seen can be found."

In the mechanical execution of the work Messrs. J. B. Lippincott & Co. have done themselves great credit. The type and paper are excellent. D. W. Y.

